# Not Home Alone: Leveraging Telehealth and Informatics to Create a Lean Model for COVID-19 Patient Home Care

**DOI:** 10.1089/tmr.2021.0020

**Published:** 2021-10-28

**Authors:** Dee Ford, Emily Warr, Cheryl Hamill, Wenjun He, Ekaterina Pekar, Jillian Harvey, Ragan DuBose-Morris, Kimberly McGhee, Kathryn King, Leslie Lenert

**Affiliations:** ^1^ Department of Medicine, Division of Pulmonary and Critical Care Medicine, College of Medicine, Medical University of South Carolina, Charleston, South Carolina, USA.; ^2^ Center for Telehealth Department, Medical University of South Carolina, Charleston, South Carolina, USA.; ^3^ South Carolina Clinical and Translational Research Institute Department, College of Medicine, Medical University of South Carolina, Charleston, South Carolina, USA.; ^4^ Department of Medicine, Information Solutions, Medical University of South Carolina, Charleston, South Carolina, USA.; ^5^ Department of Healthcare Leadership and Management, College of Health Professions, Medical University of South Carolina, Charleston, South Carolina, USA.; ^6^ Academic Affairs, Medical University of South Carolina, Charleston, South Carolina, USA.; ^7^ Department of Pediatrics, College of Medicine, Medical University of South Carolina, Charleston, South Carolina, USA.; ^8^ Department of Medicine, College of Medicine, Medical University of South Carolina, Charleston, South Carolina, USA.

**Keywords:** telehealth, telemedicine, remote patient monitoring, COVID-19

## Abstract

In response to the emerging COVID-19 public health emergency in March 2020, the Medical University of South Carolina rapidly implemented an analytics-enhanced remote patient monitoring (RPM) program with state-wide reach for SARS-CoV-2-positive patients. Patient-reported data and other analytics were used to prioritize the sickest patients for contact by RPM nurses, enabling a small cadre of RPM nurses, with the support of ambulatory providers and urgent care video visits, to oversee 1234 patients, many of whom were older, from underserved populations, or at high risk of serious complications. Care was escalated based on prespecified criteria to primary care provider or emergency department visit, with 89% of moderate- to high-risk patients treated solely at home. The RPM nurses facilitated the continuity of care during escalation or de-escalation of care, provided much-needed emotional support to patients quarantining at home and helped find medical homes for patients with tenuous ties to health care.

## Introduction

Early in the COVID-19 pandemic, reports from China,^1^ Italy,^2^ and the U.S. northeast^3^ chronicled the immense pressure that COVID-19 was placing on hospitals and health systems. The influx of COVID-19 patients to emergency departments (EDs) threatened to spread the infection to health care workers and non-COVID-19 patients.^2^ Hospitals burned through personal protective equipment at a fast clip,^4^ and hospital and especially Intensive Care Unit beds were in short supply.^5^ To address this emergency, health institutions across the globe turned to telehealth,^6^ which is recognized as essential to medical responses to disasters.^7^ Remote patient monitoring (RPM) programs, in which patients' symptoms and vital signs are sent electronically to remote clinicians, began to be implemented worldwide to ensure that COVID-19 patients were safely monitored and treated at home,^8–10^ but the staffing demands of such programs posed a threat to their long-term sustainability.^11,12^

The Medical University of South Carolina (MUSC) Health System rapidly leveraged the expertise and resources of its Center for Telehealth and Biomedical Informatics Center (BMIC), which have a long history of collaboration, to develop a scalable sustainable COVID-19 RPM program. The program, which preferentially enrolls high-risk patients and those with tenuous ties to care, has reduced the pressure on hospitals and EDs by directing only those patients with worsening symptoms to these resources. It has also provided increased surveillance, care navigation, and emotional support for patients quarantining at home, all while adopting a lean staffing model that helps ensure its sustainability.

## Rapid Response Implementation

On March 17, 2020, 11 days after the report of the first two suspected cases of COVID-19 in South Carolina, Telehealth and BMIC leaders began to discuss establishing a bioinformatics-enhanced COVID-19 RPM program. Less than 2 weeks later, on March 30, the program launched.

This rapid launch can be attributed to the strong relationship between Telehealth and BMIC leaders, who had already collaborated to expand COVID-19 screening and testing. Virtual visits, originally intended for minor medical issues, had been adapted to screen patients with possible COVID-19^13^ and refer them for SARS-CoV-2 testing at MUSC Health-affiliated mobile testing sites.^14^ BMIC created a registry of all patients who had ever tested positive for COVID-19 at an MUSC Health-affiliated mobile testing site, urgent or ambulatory care facility, or hospital. The RPM program was intended to round out these screening and testing efforts by monitoring SARS-CoV-2-positive patients quarantining at home and, when necessary, help them transition to a higher level of care. In addition, rapid implementation and patient enrollment were facilitated through the patients' use of their own thermometers and home equipment. Patients purchased home pulse oximeters or utilized built-in phone sensors to track their oxygen saturation levels.

## An Informatics-Grounded Approach

The BMIC team created and embedded a short COVID-19 REDCap^15^ questionnaire called the Symptom Checker (Table 1) into the patient portal of Epic, the electronic health record (EHR) system. The content of the questionnaire was adapted by MUSC Health clinical experts from a validated pneumonia instrument.^16^ All patients tested at an MUSC Health-sponsored site are assigned a medical record number and receive an email inviting them to sign up for the patient portal. Those who completed the process and are enrolled in the RPM program receive a daily reminder to log in to their patient portal and fill out the COVID-19 symptom questionnaire. To avoid deepening the digital divide,^17–19^ nurses do not turn away patients who are unable or unwilling to access the patient portal but instead call them to obtain information on their condition and fill out the questionnaire on their behalf.

**Table 1.** COVID-19 Remote Patient Monitoring Patient Daily Symptom Survey

No.	Question	Options
1	Are you today bothered by shortness of breath when sitting still?	Yes/No
2	Are you today bothered by shortness of breath when walking around the house/ward?	Yes/No
3	Are you today bothered by shortness of breath when washing/dressing?	Yes/No
4	Are you today bothered by shortness of breath when walking in the street?	Yes/No
5	Are you today bothered by shortness of breath when taking a shower?	Yes/No
6	Are you today bothered by shortness of breath when walking the stairs?	Yes/No
7	Rate the severity of your shortness of breath at the moment?	Not at all short of breath Slightly short of breath Fairly short of breath Substantially short of breath Terribly short of breath
8	Do you cough?	No Only in the morning, when getting up Now and then, all through the day Now and then, all through the day Frequently, all through the day
8a	Do you cough up sputum? (amount of sputum by 24 h)	No <2 spoons >2 spoons Half a cup or more
8b	Do you cough up the sputum with ease?	Not bothered by sputum With ease Fairly difficult Very difficult
8c	What is the color of the sputum?	Did not pay attention/no sputum Transparent White Green, yellow, or brown
9	How fit do you feel at the moment?	Very fit Fit Not fit
10	Express your general state of health at the moment	Excellent Good Fair Poor Very poor
11	Please describe any other important symptoms	
12	Oxygen saturation	
13	Temperature	

**Fig. 1.**
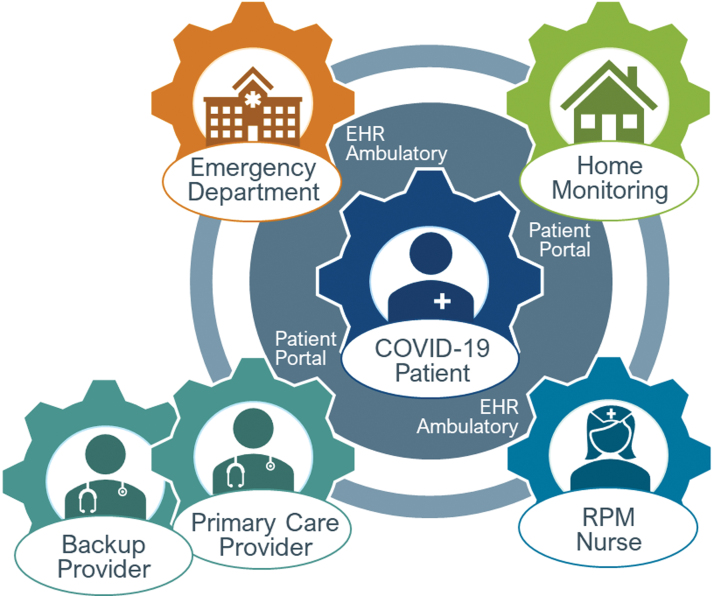
Patient-centered model for COVID-19 RPM. EHR, electronic health record; RPM, remote patient monitoring.

Patient-reported measures flow directly into the COVID-19 registry and into the EHR, where they can be accessed by RPM nurses (Fig. 1). This bioinformatics innovation has enabled RPM data to be embedded into clinical workflows, overcoming a long-standing barrier in the field.^20–22^ If a patient self-reports out-of-norm values or fails to report values for 3 consecutive days, an alert is triggered in the EHR, prioritizing that patient for review and contact by RPM nurses.

This approach enables RPM nurses to focus on patients most in need of care each day and makes possible a leaner staffing model. As a result of this data-driven targeted approach, the equivalent of five full-time RPM nurses successfully monitored and provided care navigation for 1234 COVID-19-positive patients between March 30 and the end of December 2020. This was made possible by leveraging the health system's ambulatory flexible nursing pool.

## Patient Enrollment

**Fig. 2.**
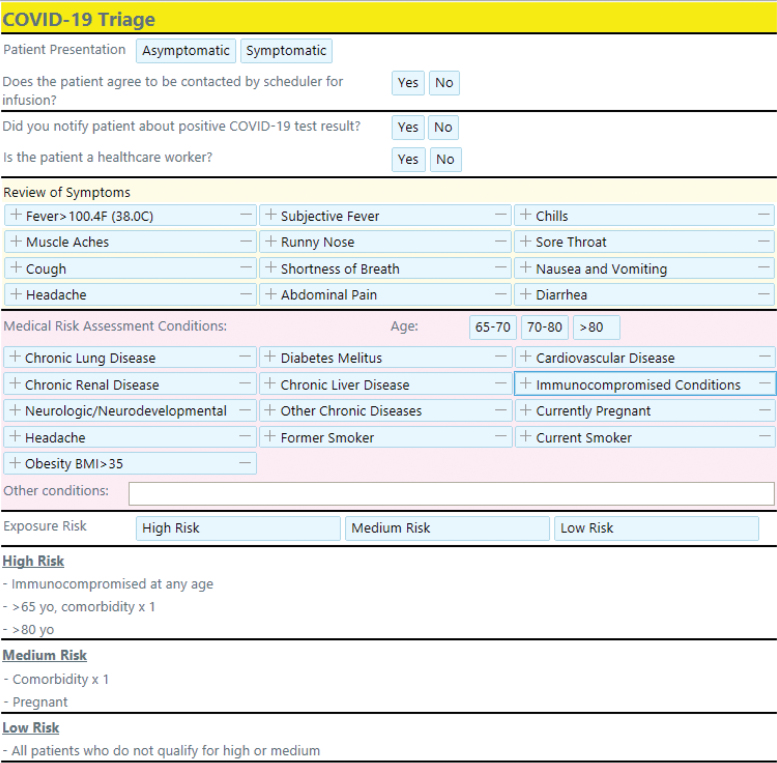
Patient triage intake form in Epic™ (Epic Systems Corporation), the EHR currently used at the MUSC. MUSC, Medical University of South Carolina.

The program preferentially enrolls patients with the highest risk of complications or the most tenuous ties to care. After reviewing registry data, RPM nurses call patients to obtain further information about their symptoms and health history. This information is entered into a BMIC-created EHR-embedded COVID-19 triage intake form (Fig. 2), which uses an algorithm created by MUSC Health clinicians to assign a risk level (low, medium, or high) for COVID-19 complications. The RPM nurse can manually override the risk assessment. Initially, high-risk patients included those who were immunocompromised, 65 years, or older with a comorbidity, or older than 80 years. Later, a moderate-to-high risk was assigned to patients aged 65 years or older, regardless of comorbidity. A body mass index of 35 kg/m^2^ or greater was also considered a major risk factor and was added to the triage form as a health condition. The patient enrollment period is based on Centers for Disease Control and Prevention guidelines. Patients are enrolled in the program for the 10–20 days that align with their home quarantine period.

## Care Pathways

**Fig. 3.**
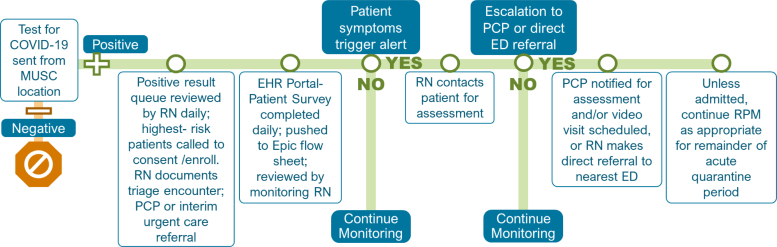
COVID-19 RPM workflow. ED, emergency department; PCP, primary care provider; RN, registered nurse.

The program's care pathways are grounded in the patient medical home and ensure continuity for patients as they transition between levels of care (Fig. 3). RPM nurses receive an alert if a patient reports being short of breath at rest, having an uncontrolled fever (>101.4°F), having low oxygen saturation levels (<95%), or having a poor health status overall. An RPM nurse will then call to obtain further information and decide whether to escalate to a primary care provider (PCP) visit, often through video visit. Through the registry, RPM nurses have access to PCP contact information. The PCP will determine whether the treatment needs to be adjusted or whether referral to the ED is warranted. For patients without a PCP, an affiliated urgent care network provides PCP visits through video during the quarantine period. These visits are free of charge and are available same day for urgent matters and within 3 days for continuum of care issues.

For the most serious cases, RPM nurses, who can access information about local ED capacity through the EHR, directly refer patients to an ED. RPM nurses follow-up to learn whether patients have been seen in an ED and/or admitted. When hospitalized, patients are disenrolled from the RPM program but are often re-enrolled upon discharge for continued monitoring during the remainder of the quarantine period. The program also offers RPM postdischarge for any patient who has been treated for COVID-19 at any MUSC Health hospital.

## Program Metrics

### Effective care navigation for at-risk patients

**Fig. 4.**
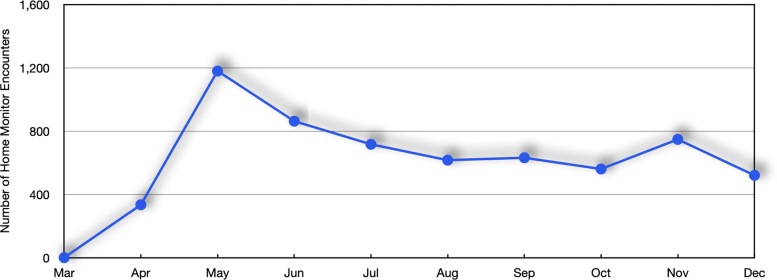
Number of home monitoring encounters by month.

From March 30 to late December 2020, 1234 out of 19,293 patients who had ever tested positive at an MUSC-sponsored site had enrolled in the RPM program. Table 2 provides demographic and risk stratification groupings for enrolled patients. Of note, 75% were at moderate-to-high risk of serious complications, and the proportion of under-represented minorities enrolled was substantially higher than regional demographics (49.8% in the RPM program vs. 37.4% for the state of South Carolina).^23^ During that period, a total of 6165 home monitoring encounters occurred, with the highest number occurring in July, which coincided with a local surge in cases (Fig. 4). The number of encounters per patient ranged from 3.8 to 6.0 from April to December 2020, with a standard deviation of 0.7.

**Table 2.** Characteristics and Referral Data for 1234 Patients Enrolled in COVID-19 Remote Patient Monitoring Program

Patient characteristics	n (%)
Age, mean (SD)	55.3 (17.2)
Gender	
Female	789 (63.9)
Male	436 (35.3)
Unknown	9 (0.7)
Race	
American Indian of Alaska Native	4 (0.3)
Asian	6 (0.5)
Black or African American	582 (47.2)
Hispanic or Latino	22 (1.8)
White	524 (42.6)
Two or more	1 (0.1)
Other	30 (2.4)
Unknown/refused	65 (5.3)
Uninsured	224 (18.2)
No PCP	301 (24.4)
Active patient portal account	917 (74.3)
Risk^a^	
Low risk	312 (25.4)
Medium risk	477 (38.8)
High risk	439 (35.8)
Referral and re-enrollment	
Referral by RPM to PCP visit	445 (36.1)
Referral by RPM to ED	124 (10.0)
Re-enrolled with RPM after discharge	7 (0.6)

^a^ The denominator used to calculate risk percentages was 1228.

ED, emergency department; PCP, primary care provider; RPM, remote patient monitoring; SD, standard deviation.

RPM nurses referred 445 (36%) patients for a visit with their PCP and 124 (10%) directly to the ED. Seven were re-enrolled into the RPM program upon discharge from the hospital for continued monitoring to ensure good continuity of care. In aggregate, 89% of the 916 patients at moderate or high risk of severe complications were managed solely at home.

### Patient satisfaction

**Fig. 5.**
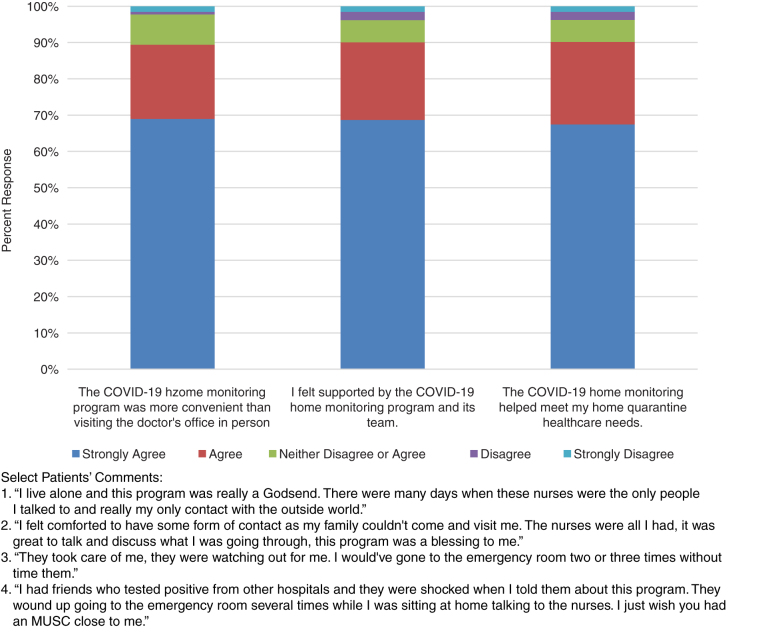
Patient satisfaction.

Upon completion of the RPM program, patients are asked to assess their program experience by completing a Redcap survey, which they can access through their patient portal or email. An optional follow-up personalized telephone interview is also offered. To date, 132 patients have responded to the emailed survey, and 61 have also provided detailed information during the follow-up call. Across all survey questions, 90% of patients report high program satisfaction (agree or strongly agree). Patients indicate feeling isolated during COVID-19 and emphasize that the program provided reassurance and guidance. Many of those who signed up for the patient portal appreciated the two-way communication it afforded them with RPM nurses and other providers. Figure 5 summarizes patient experience responses and program feedback.

### Challenges and program evolution

Patient and provider feedback has guided the program's evolution. When patients requested two-way communication with staff, an additional “free” text field for messages was added into the Symptom Checker. Patient feedback also led to creation of a process for obtaining work excuses and back-to-work clearances through the patient portal. Nurses' needs were addressed with continuously updated workflows, tip sheets, and training materials and better data visualization tools.

Initially, we relied on PCPs with a background in telehealth and/or health disparities when escalation of care was required. As we scaled the program, it became clear that basing it in the patient medical home provided better continuity of care while also reducing program staffing needs. For patients without a PCP, an affiliated urgent care network provides PCP backup through video visit.

We have had to guard against “mission creep,” which can set in when patients who do not have PCPs try to meet their non-COVID-19-related medical needs through the program. We are addressing this issue by rigorously maintaining the program's focus on COVID-19, while also helping patients without a tie to primary care to establish a relationship with a local PCP or virtual provider.

Our program did not systematically identify patients for whom physiologic monitoring (e.g., home pulse oximetry) might have value and did not offer such monitoring. We have developed infrastructure to include these biometric measures, but the need for rapid response and unclear patient benefit ultimately guided us toward patient-reported measures as our primary focus.

## Next Steps and Conclusions

More robust patient dashboards are being developed for improved RPM nurse efficiency. In close collaboration with PCPs, the program will offer extended monitoring of patients with “long haul” COVID-19. Currently, our health system offers this program without cost to patients and payors. However, this is unsustainable, and we are developing pathways to capture revenue associated with RPM programs appropriately.^24,25^

The COVID-19 RPM program at MUSC has enabled a small cadre of RPM nurses, with the support of the ambulatory flex nursing pool and video visits provided by either their established PCP or an affiliated urgent care network, to offer at-home care for patients with COVID-19. In close collaboration with patients' PCPs, the RPM nurses have been able to escalate care, referring to EDs when necessary, and to continue monitoring patients postdischarge to ensure continuity of care. The program has provided much-needed emotional and other support to patients quarantining at home and has encouraged increased patient portal uptake. Finally, it has helped connect patients without PCPs to primary care for the first time, which could have lasting benefits for their health.

## Abbreviations Used

BMIC: Biomedical Informatics Center
ED: emergency department
EHR: electronic health record
MUSC: Medical University of South Carolina
PCP: primary care provider
RPM: remote patient monitoring.
SD: standard deviation

## References

[1] Wu Z, McGoogan JM. Characteristics of and important lessons from the coronavirus disease 2019 (COVID-19) outbreak in China: summary of a report of 72 314 cases from the Chinese Center for Disease Control and Prevention. JAMA 2020;323:1239–1242. 32091533 10.1001/jama.2020.2648

[2] Nacoti M, Ciocca A, Giupponi A, et al. At the epicenter of the Covid-19 pandemic and humanitarian crises in Italy: changing perspectives on preparation and mitigation. NEJM Catalyst Innov Care Deliv [Epub ahead of print]; https://catalyst.nejm.org/doi/full/10.1056/CAT.20.0080

[3] Jeffery MM, D'Onofrio G, Paek H, et al. Trends in Emergency Department Visits and Hospital Admissions in Health Care Systems in 5 States in the First Months of the COVID-19 Pandemic in the US. JAMA Intern Med 2020;180:1328–1333. 32744612 10.1001/jamainternmed.2020.3288 PMC7400214

[4] Ranney ML, Griffeth V, Jha AK. Critical Supply Shortages—The Need for Ventilators and Personal Protective Equipment during the Covid-19 Pandemic. N Engl J Med 2020;382:e41. 32212516 10.1056/NEJMp2006141

[5] Blavin F, Arnos D. Hospital Readiness for COVID-19: analysis of bed capacity and how it varies across the country. State Coverage Initiat Issue Brief 2020. https://www.rwjf.org/en/library/research/2020/03/hospital-readiness-for-covid19-analysis-of-bed-capacity-and-how-it-varies-across-the-country.html

[6] Wosik J, Fudim M, Cameron B, et al. Telehealth transformation: COVID-19 and the rise of virtual care. J Am Med Inform Assoc 2020;27:957–962. 32311034 10.1093/jamia/ocaa067 PMC7188147

[7] Lurie N, Carr BG. The role of telehealth in the medical response to disasters. JAMA Intern Med 2018;178:745–746. 29710200 10.1001/jamainternmed.2018.1314

[8] Behar J, Liu C, Kotzen K, et al. Remote health diagnosis and monitoring in the time of COVID-19. Physiol Meas 2020;41:10TR01. 10.1088/1361-6579/abba0a PMC9364387 32947271

[9] Halberthal M, Nachman D, Eisenkraft A, Jaffe E. Hospital and home remote patient monitoring during the COVID-19 outbreak: a novel concept implemented. Am J Disaster Med 2020;15:149–151. 32804396 10.5055/ajdm.2020.0349

[10] Shaw JG, Sankineni S, Olaleye CA, et al. A novel large scale integrated telemonitoring program for COVID-19. Telemed J E Health [Epub ahead of print]; DOI: 10.1089/tmj.2020.0384 33544043

[11] Kricke G, Roemer PE, Barnard C, Peipert JD, Henschen BL, Bierman JA, Blahnik D, Grant M, Linder JA. Rapid implementation of an outpatient Covid-19 monitoring program. NEJM Catalyst Innov Care Deliv 2020; https://catalyst.nejm.org/doi/pdf/10.1056/CAT.20.0214.

[12] Annis T, Pleasants S, Hultman G, et al. Rapid implementation of a COVID-19 remote patient monitoring program. J Am Med Inform Assoc 2020;27:1326–1330. 32392280 10.1093/jamia/ocaa097 PMC7239139

[13] Ford D, Harvey JB, McElligott J, et al. Leveraging health system telehealth and informatics infrastructure to create a continuum of services for COVID-19 screening, testing, and treatment. J Am Med Inform Assoc 2020;27:1871–1877. 32602884 10.1093/jamia/ocaa157 PMC7337763

[14] Obeid JS, Davis M, Turner M, et al. An artificial intelligence approach to COVID-19 infection risk assessment in virtual visits: a case report. J Am Med Inform Assoc 2020;27:1321–1325. 32449766 10.1093/jamia/ocaa105 PMC7313981

[15] Harris PA, Taylor R, Thielke R, Payne J. Research electronic data capture (REDCap)—a metadata-driven methodology and workflow process for providing translational research informatics support. J Biomed 2009;42:377–381. 10.1016/j.jbi.2008.08.010 PMC2700030 18929686

[16] Moussaoui RE, El Moussaoui R. Development and validation of a short questionnaire in community acquired pneumonia. Thorax 2004;59:591–595. 15223867 10.1136/thx.2003.015107 PMC1747065

[17] Mackert M, Mabry-Flynn A, Champlin S, Donovan EE, Pounders K. Health literacy and health information technology adoption: the potential for a new digital divide. J Med Internet Res 2016;18:e264. 27702738 10.2196/jmir.6349 PMC5069402

[18] Ray KN, Kahn JM. Connected subspecialty care: applying telehealth strategies to specific referral barriers. Acad Pediatr 2020;20:16–22. 31404707 10.1016/j.acap.2019.08.002 PMC6944761

[19] Anthony DL, Campos-Castillo C, Lim PS. Who isn't using patient portals and why? Evidence and implications from a national sample of US adults. Health Affairs 2018;37:1948–1954. 30633673 10.1377/hlthaff.2018.05117

[20] Abdolkhani R, Gray K, Borda A, DeSouza R. Patient-generated health data management and quality challenges in remote patient monitoring. JAMIA Open 2019;2:471–478. 32025644 10.1093/jamiaopen/ooz036 PMC6993998

[21] Cohen DJ, Keller SR, Hayes GR, Dorr DA. Integrating patient-generated health data into clinical care settings or clinical decision-making: lessons learned from project healthdesign. JMIR Human 2016;3:e26. 10.2196/humanfactors.5919 PMC5093296 27760726

[22] Gandrup J, Ali SM, McBeth J, van der Veer SN, Dixon WG. Remote symptom monitoring integrated into electronic health records: a systematic review. J Am Med Inform Assoc 2020;27:1752–1763. 32968785 10.1093/jamia/ocaa177 PMC7671621

[23] United States Census Bureau. QuickFacts: South Carolina. Available at https://www.census.gov/quickfacts/fact/table/SC/RHI225219 Accessed 1 April, 2021.

[24] Wechsler LR, Adusumalli S, Deleener ME, Huffenberger AM, Kruse G, Hanson CW III. Reflections on a Health System's Telemedicine Marathon. Telemed Rep 2020;1:2–7. 10.1089/tmr.2020.0009 PMC8812293 35722251

[25] Thomas EE, Haydon HM, Mehrotra A, et al. Building on the momentum: sustaining telehealth beyond COVID-19. J Telemed Telecare [Epub ahead of print]; DOI: 10.1177/1357633X20960638 32985380

